# Blockade of Axl signaling ameliorates HPV16E6-mediated tumorigenecity of cervical cancer

**DOI:** 10.1038/s41598-017-05977-8

**Published:** 2017-07-18

**Authors:** Eun-Hee Lee, Kon-Young Ji, Eun-Mi Kim, Su-Man Kim, Hyeong-Woo Song, Ha-Rim Choi, Byung Yeoup Chung, Hyo Jin Choi, Hyoung-Woo Bai, Hyung-Sik Kang

**Affiliations:** 1 Research Division for Biotechnology, Advanced Radiation Technology Institute (ARTI), Korea Atomic Energy Insitute (KAERI), 29 Geumgu-gil, Jeongeup-si, Jeollabuk-do 580-185 Korea; 2 Predictive Model Research Center, Korea Institute of Toxicology, 141 Gajeong-ro, Yuseong-gu, Daejeon 34114 Republic of Korea; 3 School of Biological Sciences and Technology, Chonnam National University, 77 Yongbong-ro, Buk-gu, Gwangju 500-757 Korea; 4 Department of Nursing, Nambu University, 23 Chumdan Jungang-ro, Gwangsan-gu, Gwangju 506-706 Korea

## Abstract

Axl receptor tyrosine kinase is involved in the tumorigenesis and metastasis of many cancers. Axl expression was markedly higher in human papilloma virus type 16E6 (HPV16E6)-overexpressing HeLa (HE6F) cells and lower in HPV16E6-suppressing CaSki (CE6R) cells than in the controls. SiRNA-mediated knockdown of E6 expression led to increased phosphatase and tensin homolog (PTEN) phosphorylation at Ser380 and attenuated AKT phosphorylation. Expression of membrane-associated guanylate kinase inverted-2 (MAGI-2), an E6-induced degradation target, was induced in E6-siRNA-transfected cells. Moreover, myeloid zinc finger protein 1 (MZF1) binds directly to the *Axl* promoter in HE6F cells. Axl expression was regulated by HPV16E6-mediated PTEN/AKT signalling pathway, and *Axl* promoter activity was regulated through MZF1 activation in cervical cancer, which promoted malignancy. Axl silencing suppressed the metastasis of Caski cells and enhanced the susceptibility to NK cell-mediated killing of HE6F cells. In addition, the expression of Axl and MZF1 was highly correlated with clinical stage of cervical cancer and HPV16/18 infection. Taken together, Axl expression was induced by HPV16E6 in cervical cancer cells, suggesting that blockade of Axl signalling might be an effective way to reduce the progression of cervical cancer.

## Introduction

Human papillomaviruses (HPVs) are small, non-enveloped viruses that induce squamous epithelial tumours^1^. HPV16 and 18 are the most prevalent types and are associated with genomic integration and cervical cancer^2, 3^. The HPV-encoded oncoproteins, namely E6 and E7, induce the immortalization and transformation of the primary cervical keratinocytes that are involved in tumour progression^4^. The E6 oncoprotein inactivates the tumour suppressor p53 by binding in a complex with ubiqutin ligase E6AP and p53, leading to ubiquitination and degradation of p53. The E7 oncoprotein binds to retinoblastoma (RB) gene family proteins, resulting in the upregulation of proteins associated with cell cycle progression^5^. Collectively, the expression of the HR-HPV E6/E7 genes directly contributes to malignant progression by subverting genomic stability, which is also necessary for the induction of premalignant alterations. Interestingly, a unique feature of the HPV E6 oncoproteins is the presence of a PDZ (PSD-95⁄DLG⁄ZO-1) binding motif (PBM) at their C-termini. Through this motif, E6 can interact with a large number of cellular proteins that contain PDZ domains, which play a critical role in anchoring proteins to the cell surface. HPV E6 proteins have several cellular binding partners, including the human homologues of Drosophila disc large (DLG), hDLG, Scribble, MUPP1, membrane-associated guanylate kinase inverted-1 (MAGI-1), MAGI-2, and MAGI-3^6^. Among them, it is known that MAGI-2 enhances the ability of PTEN to suppress AKT activation through an interaction between the PDZ-binding motif of PTEN and the second PDZ domain of MAGI-2^7^. Despite the increasing significance of E6-associated oncogenic events in transformed human cells, the molecular events that govern their regulation of cellular oncogene expression remain unclear.

Axl belongs to a receptor tyrosine kinase that was originally isolated from chronic myeloid leukaemia patients^8^. Growth arrest specific gene 6 (Gas6) is a common ligand of Axl. Abnormal expression and over-activation of Axl protein protect cells from apoptosis and increase migration, aggregation, and growth in several human solid cancers^9–14^. Axl expression correlates with poor prognosis in patients with acute myeloid leukaemia^15^, gastric^16^ and advanced lung adenocarcinoma^14^ and with the adhesion, motility, and invasiveness of osteosarcoma cells^17^. Axl-dependent signal transduction has been implicated in the modulation of tumour invasion and metastasis through activation of PI3K and its downstream target, serine/threonine protein kinase AKT (AKT), as a central step^18^. A recent report suggested that MZF1 induces migration, invasion, and *Axl* gene expression in cervical cancer cells^19^. Furthermore, another study demonstrated that MZF1 is involved in the E6-mediated Foxhead box M1 (FOXM1)/NKX2-1 expression through the Wnt/β-catenin signalling pathway, resulting in tumour progression and poor outcomes in HPV-positive patients^20^. However, the exact external signals that regulate the transcriptional activity of the MZF1 transcription factor by E6 remain unclear. Moreover, although Axl is correlated with tumour progression and poor prognosis in various human carcinomas, the underlying molecular and cellular mechanisms associated with E6 oncoprotein are still unknown.

Here, we demonstrated a novel molecular mechanism by which HPV type 16 E6 oncoprotein induced Axl expression through the MAGI-2/PTEN/AKT signalling pathway in human cervical cancer cells. Axl silencing inhibited the metastasis of Caski cells and enhanced NK cell-mediated killing of HE6F cells. Therefore, Axl acts as a risk factor for the development of cervical cancer by suppressing immunity and inducing evasion from host defense system. Our findings suggest that blockade of Axl signaling is required for the suppression of HPV16E6-mediated progression of cervical cancer.

## Results

### HPV16E6 oncoprotein induces Axl expression

**Figure 1:**
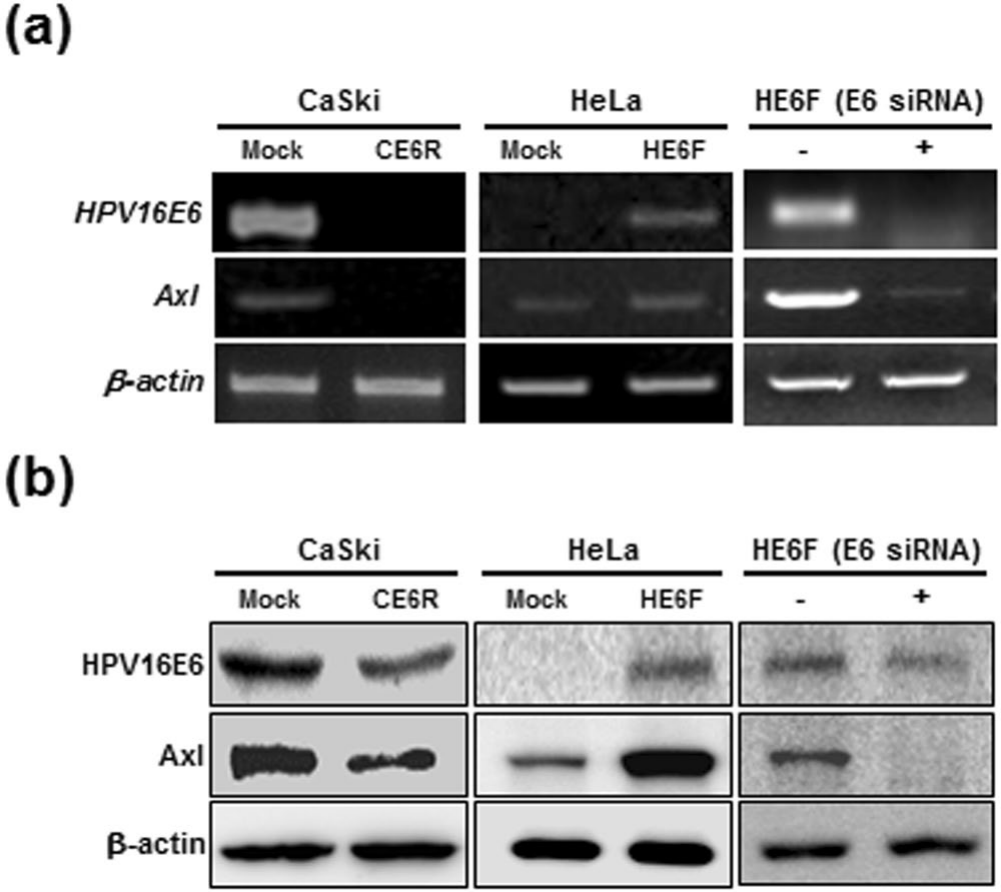
HPV16E6 upregulates expression of Axl. (**a** and **b**) CE6R and HE6F cells were transfected with pcDNA-*HPV16E6* (containing antisense or sense HPV16E6 RNA) and ectopic expression of HPV16E6 and Axl were confirmed by RT-PCR (upper panel) and western blotting (lower panel). HE6F cells transfected with HPV16E6 siRNA were used to confirm the expression of E6 and Axl by RT-PCR (upper panel, right) and western blotting (lower panel, right). β-Actin and α-tubulin were used as internal controls, and the data are representative of at least five independent experiments.

To investigate the potential effect of E6 on Axl expression, we examined Axl expression by reverse transcription (RT)-PCR and western blot analysis in E6 stable transfectants. We established a HeLa cell line (HPV-18 positive) stably expressing HPV16E6 (HE6F) and a CaSki cell line (HPV-16 positive) stably expressing HPV16E6 antisense RNA (CE6R)^21^. We found that HE6F cells showed higher *Axl* mRNA and protein levels than mock control cells. As expected, Axl expression was diminished in CE6R cells. Then, we examined whether Axl expression in HE6F cells was decreased by transfection with an HPV16E6-specific siRNA. Likewise, knockdown of HPV16E6 diminished *Axl* transcription levels in HE6F cells (Fig. 1a and b). The decrease in *Axl* mRNA levels was correlated with the down-regulation of Axl protein expression in these cells. Taken together, these results suggest that Axl expression is upregulated by E6 in cervical cancer cells.

### MZF1 transactivates the *Axl* promoter

**Figure 2:**
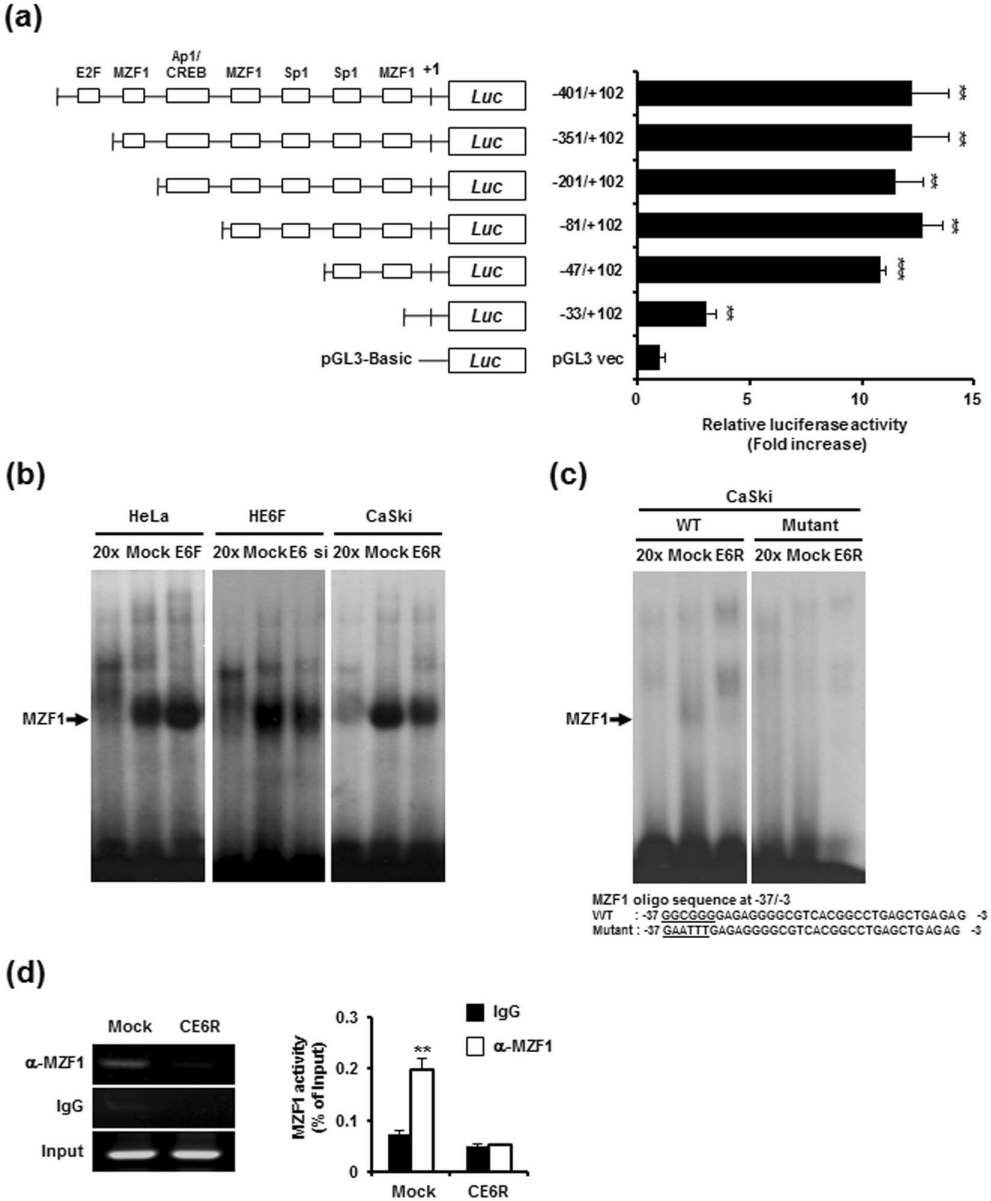
Transcriptional activation of MZF1 in the regulation of E6-mediated Axl expression. (**a**) Luciferase assays of lysates from 293 T cells following co-transfection with *Axl* promoter deletion constructs and a plasmid encoding HPV16E6 as indicated. Data are the mean ± s.e.m. from three independent experiments (*P < 0.05; **P < 0.01; ***P < 0.001). (**b**) MZF1 DNA binding ability was analysed by EMSA using nuclear extracts obtained from HE6F (left), E6-silenced HE6F (middle), and CE6R (right) cells and corresponding mock controls. Oct-1 was used as an internal control for equal protein loading. Cold 20 × contains a 20-fold excess of unlabelled MZF1 probe for competition analysis. (**c**) EMSA was done with oligonucleotides specific for MZF1 binding motif at −37/−3 bp or with mutated sequence of MZF1. (**d**) DNA-protein complexes were immunoprecipitated from cross-linked chromatin using antibody specific for MZF1 and normal IgG control. The immunoprecipitated chromatin was subjected to PCR analysis using the indicated primers (left), and the binding activity was represented as a bar graph after calculating a relative percentage compared with corresponding levels in input DNA (right panel). The graphs represent the mean ± standard deviation of triplicate determinations (**P < 0.01).

The *Axl* promoter region is a GC-rich sequence that lacks typical TATA and CAAT boxes. However, this GC-rich region harbours multiple MZF1 and SP transcription factor binding sites^19^. To identify the transcription factor involved in E6-mediated *Axl* promoter activation, deletions in the promoter proximal region were constructed and transiently co-transfected with pcDNA3.1-E6 into 293 cells (Fig. 2a). A luciferase reporter driven by the −401/+102 upstream region of *Axl*, which contains multiple transcription factor binding regions, was used. Using a cotransfection assay, a robust reduction in *Axl* promoter activity was observed in a mutant with a deletion of the MZF1 element in the −47/+102 region, suggesting that this MZF1 element inhibited E6-induced *Axl* promoter activity. To verify that the transcriptional activity of MZF1 at the *Axl* promoter is regulated by E6, an electrophoretic mobility shift assay (EMSA) was performed in E6-silenced and E6-overexpressing transfectants (Fig. 2b). An EMSA analysis using MZF1-consensus oligonucleotides demonstrated that MZF1 binding to specific oligonucleotides was increased in HE6F cells and suppressed in CE6R cells and E6-silenced HE6F cells. We further performed EMSA with oligonucleotides corresponding to the *Axl* promoter region (−37/−3 bp) containing of MZF1 binding sequence (−37/−30 bp) or with mutated oligonucleotides of MZF1 binding region instead of MZF1 consensus site. WT oligonucleotides bound with MZF1 in mock control, but not in CE6R cells (Fig. 2c). The mutated oligonucleotides did not bind to MZF1 in both mock control and CE6R cells. These results suggest that MZF1 binding motif at −37/−30 bp acts as the most important motif for *Axl* transcriptional activation. Next, to verify whether MZF1 directly binds to *Axl* promoter in cervical cancer, we performed a ChIP assay using anti-MZF1 or IgG control in mock control and CE6R cells. The ChIP assay revealed that endogenous MZF1 was recruited to *Axl* promoter region in mock control, but not in CE6R cells (Fig. 2d). These data suggest that HPV16E6 drives *Axl* transcription through direct binding of MZF1 to *Axl* promoter. To determine E6 can regulate the expression of MZF1, we performed western blot analysis after transfected with E6-specific siRNAs in HE6F cells. The expression of MZF1 was decreased in E6-silenced HE6F cells, implicating that E6 may be involved in the regulation of MZF1 expression (Supplementary Fig. S1). These results suggest that MZF1 is a major element regulating Axl gene expression in HPV type16E6-infected cells.

### AKT phosphorylation and MAGI-2 protein are critical for HPV16E6-mediated Axl expression

**Figure 3:**
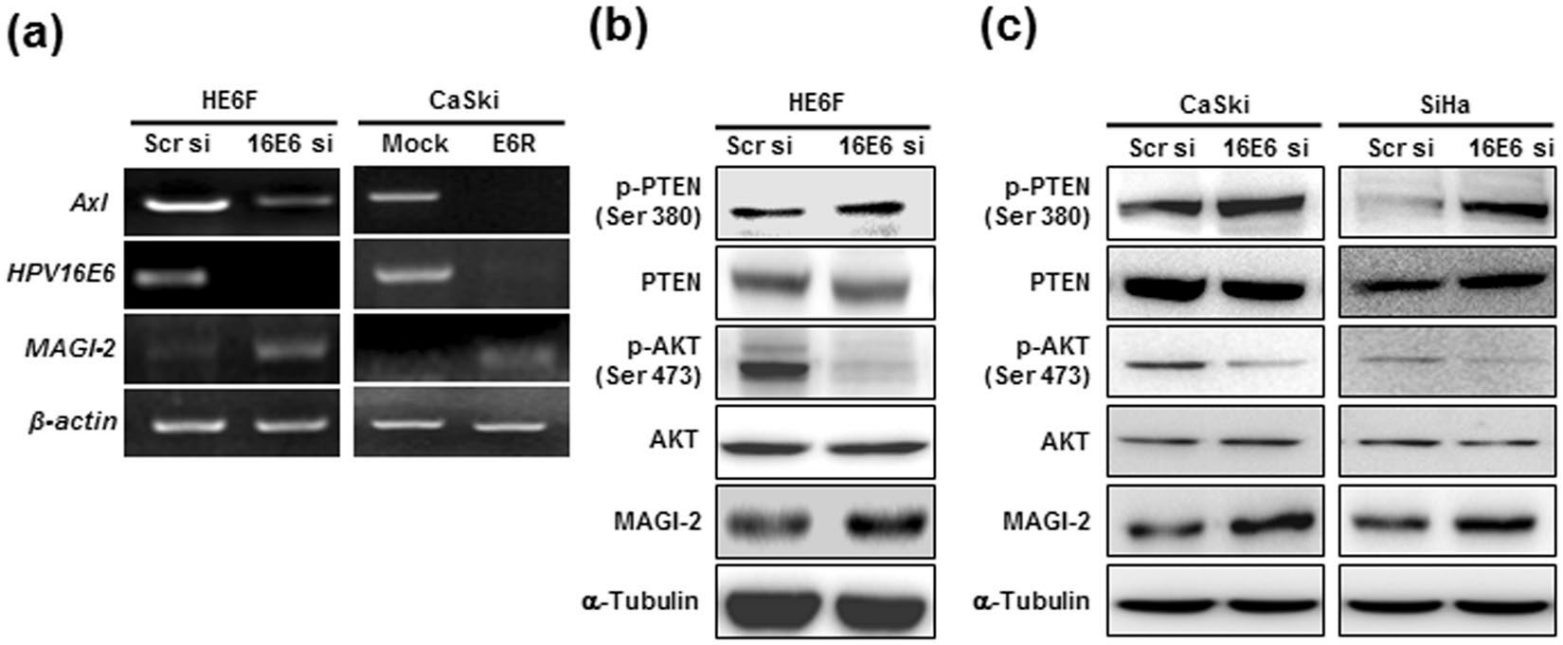
The MAGI-2/PTEN/AKT signalling pathway is involved in HPV16E6-mediated Axl expression. (**a**) MAGI-2 expression by knockdown of endogenous HPV16E6 was confirmed by RT-PCR in HE6F (left panel) and CE6R (right panel) cells compared to corresponding mock controls. (**b**,**c**) Expression of phosphorylated AKT and PTEN was analysed by western blot analysis in HPV16E6-knocked down (siRNA) HE6F (**b**), CaSki, and Siha cells (**c**). Upregulation of protein expression of MAGI-2 by HPV16E6 siRNA in HE6F, CaSki, and Siha cells was confirmed by western blotting.

Several studies have shown that E6 can activate the PI3K/AKT pathway through various mechanisms^22–24^. Oncogenic HPV16E6 proteins target MAGI-2 for degradation, resulting in a loss of PTEN activity, which regulates the PI3K/AKT pathway^25^. MAGI-2 regulates PTEN activity through its association with the PDZ binding domain, leading to increased PTEN protein stability and suppressed AKT activation. To determine whether MAGI-2/PTEN/AKT is involved in E6-mediated Axl expression, we performed RT-PCR and western blotting to examine the expression and activation status of these proteins in E6-silenced HE6F, CaSki, and Siha cells. RT-PCR analysis showed that *MAGI-2* gene expression was increased in E6-silenced HE6F cells when compared to that in the mock control (Fig. 3a, left panel). Similar results were also observed in CE6R cells (Fig. 3a, right panel). To determine whether MAGI-2 and PTEN were co-localized in cervical cancer, the immunofluorescence analysis was performed in CaSki cells. The co-localization of MAGI-2 and PTEN was observed in both mock control and CE6R cells, but much higher co-localization level in CE6R cells compared with mock control (Supplementary Fig. S3). Furthermore, PTEN phosphorylation at S380 was increased in E6-silenced HE6F cells when compared with the levels in the mock control, whereas AKT phosphorylation was diminished under these conditions (Fig. 3b). In addition, the expression of MAGI-2 protein was increased in E6-silenced HE6F cells. Similar expression patterns were observed in E6-silenced CaSki and Siha cells (Fig. 3c). Moreover, inhibition of AKT activity by Ly294002 effectively blocked E6-mediated Axl expression (Supplementary Fig. S2). Collectively, the phosphorylation of AKT at ser 473 residues was upregulated by inhibition of MAGI-2/PTEN activity due to E6 silencing in cervical cancer. These data raise the interesting possibility that MAGI-2/PTEN/AKT might be involved in the signalling pathway related to E6-mediated Axl expression.

### MZF1 and MAGI-2 are involved in HPV16E6-mediated Axl expression and Axl-mediated tumour invasion

**Figure 4:**
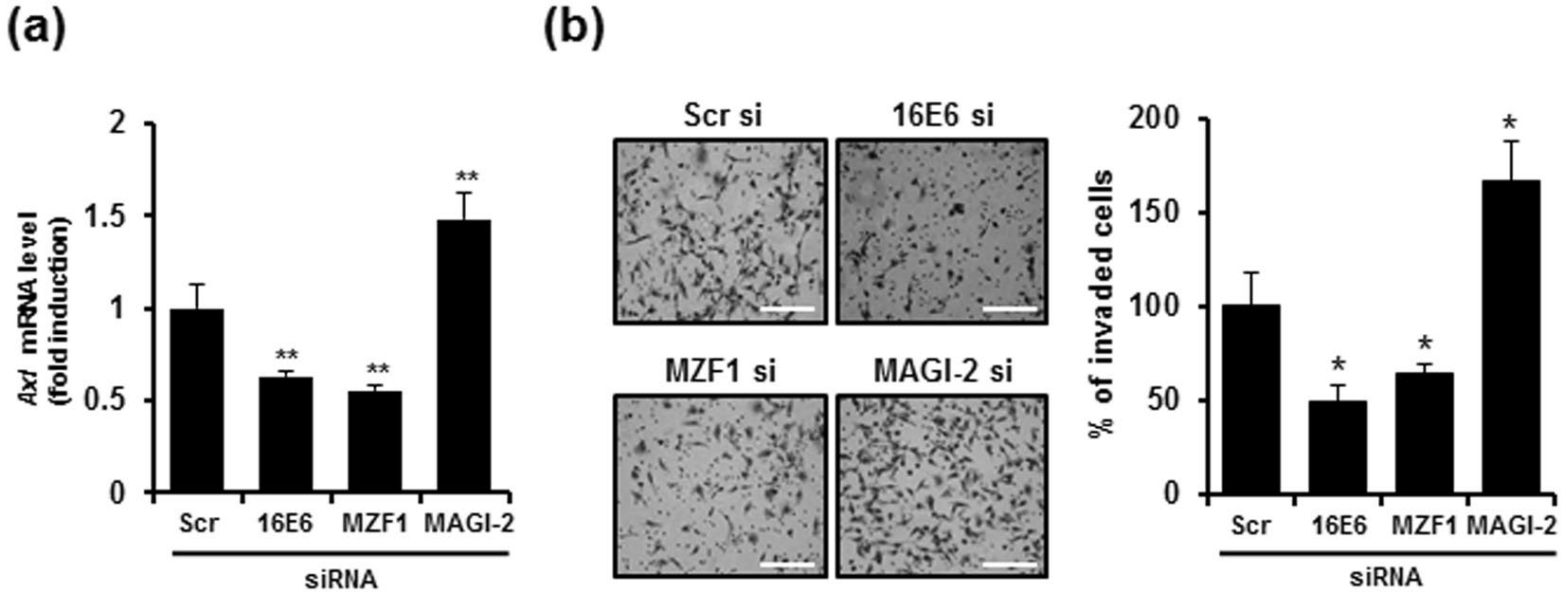
Involvement of MAGI-2 and MZF1 in E6-mediated Axl expression modulates the tumour cell invasiveness. (**a**) Axl expression levels were determined by real-time RT-PCR in HE6F cells transfected with HPV16E6, MZF1, or MAGI-2 siRNA as indicated. (**b**) Representative images of transwell invasion assay after transfection of HE6F cells with HPV16E6, MZF1, or MAGI-2 siRNA. (left panel). Scale bars, 100 μm. The percentages of invaded cells were calculated from the images in bar graph (right panel). Data in (**a)** and **(b)** are shown as the mean ± s.e.m. (*P < 0.05; **P < 0.01; ***P < 0.001). Data are representative of five independent experiments.

To elucidate whether MZF1 and MAGI-2 are involved in E6-mediated Axl expression, we performed real-time PCR to examine *Axl* gene expression in MZF1- and MAGI-2-silenced HE6F cells. We found that knockdown of E6 and MZF1 suppressed *Axl* expression by 0.4–0.5-fold in HE6F cells compared with the levels in mock control cells, whereas silencing of MAGI-2 increased *Axl* gene expression by 1.5-fold (Fig. 4a). The silencing of MAGI-2 induced upregulation of MZF1 in HE6F cells (Supplementary Fig. S4). These data suggested that MAGI-2 negatively regulated the expression of Axl by inhibiting transcriptional activation of MZF1. Previous publications reported that Axl activity is positively correlated with tumour metastasis^26, 27^. Using an invasion assay, we found that downregulation of Axl expression by silencing of E6 or MZF1 in HE6F cells highly suppressed tumour cell invasion (Fig. 4b). However, the invasiveness of MAGI-2-silenced HE6F cells was enhanced compared to that of the mock control, suggesting that E6-mediated Axl expression regulates the invasiveness of HE6F cells through MZF1 and MAGI-2.

### Blockade of Axl signaling inhibits HPV16E6-mediated tumorigenicity of cervical cancer

**Figure 5:**
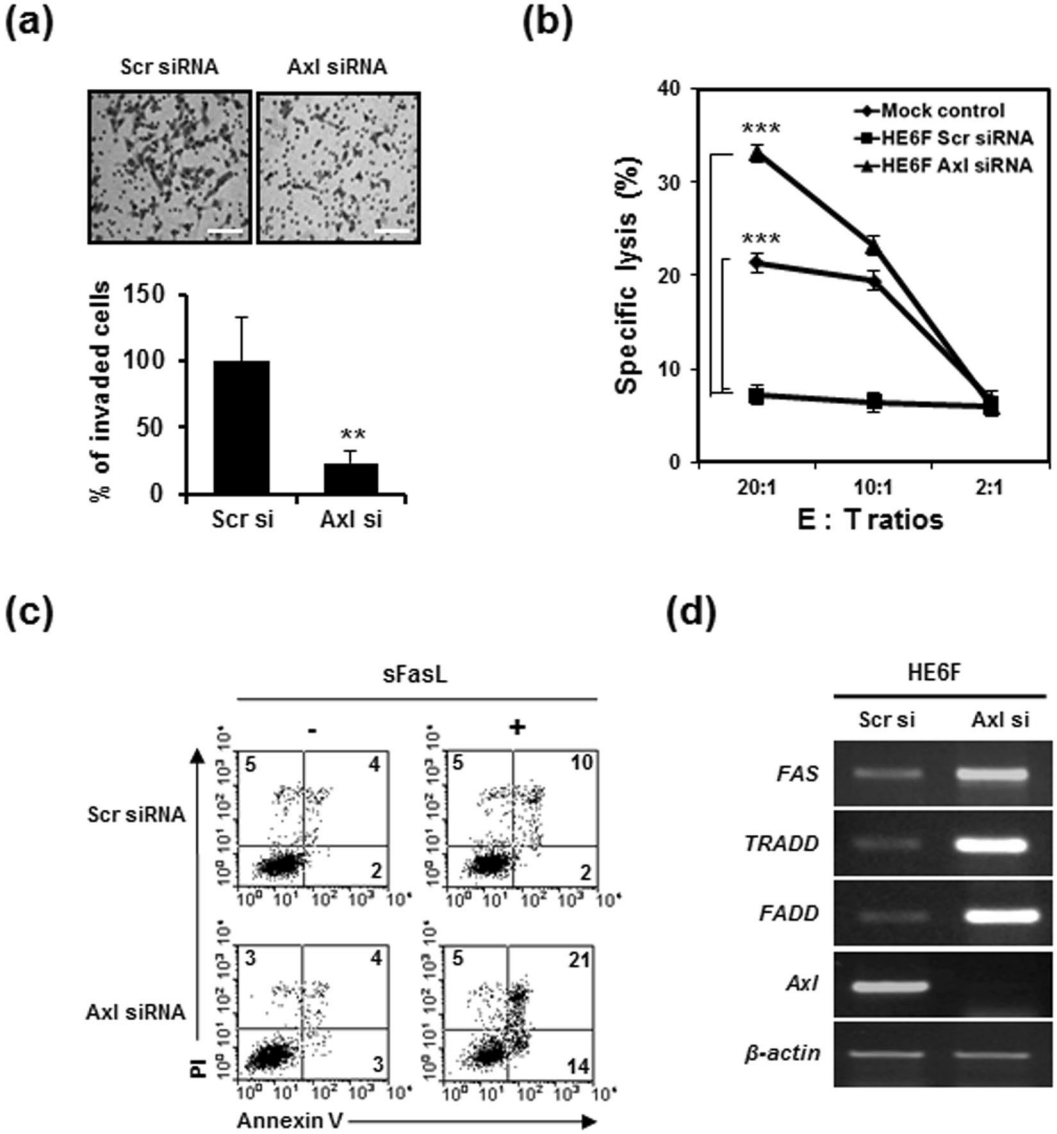
Tumorigenic effect of E6-induced Axl expression in cervical cancer cells. (**a**) Representative images of transwell invasion assay after transfection of HE6F cells with scramble and Axl siRNA (upper panel). Scale bars, 100 μm. The percentages of invaded cells were calculated from the images in bar graph (lower panel). (**b**) HPV16E6-mediated Axl expression modulates susceptibility to NK cell cytotoxicity in HE6F cells. Cytolysis activity was determined by an LDH release assay at the indicated effector:target (E:T) ratios. Data in b are shown as the mean ± s.e.m. (***P < 0.001). Data are representative of five independent experiments. (**c**) HE6F cells treated with sFasL and transfected with Axl siRNA were analysed for apoptosis by flow cytometry using annexin V-FITC and propidium iodide (PI) staining. The numbers in each quadrant represent the percentage of cells positive for annexin V and/or PI. (**d**) Expression of apoptosis-related genes was analysed by RT-PCR in Axl-silenced HE6F cells compared to scrambled siRNA-transfected cells.

Given the high expression of Axl in HE6F cells overexpressing E6, we asked whether Axl modulates the invasion, apoptosis, and susceptibility to natural killer (NK)-mediated lysis of human cervical cancer cells. E6-overexpressing HE6F cells were transfected with two distinct Axl-specific siRNAs to examine the effect of Axl on tumorigenicity in E6-overexpressing HE6F cells. Transfection of Axl-specific siRNAs specifically inhibited *Axl* gene and protein expression (Supplementary Fig. S5), and the invasiveness of HE6F cells was markedly decreased by Axl knockdown (Fig. 5a). In addition, the lytic activity of NK cells against Axl-silenced HE6F cells was increased compared with that against scrambled siRNA-transfected HE6F cells (Fig. 5b). Notably, lytic activity was highly suppressed by overexpression of E6 in HeLa cells compared with that in mock control cells. This implied that induction of Axl expression by E6 reduced the susceptibility of HeLa cells to NK-mediated lysis. To determine whether Axl plays a role in apoptosis of HE6F cells, we measured the percentage of apoptotic cells by FACS analysis after 12 h of treatment with soluble Fas ligand (sFasL) in Axl-silenced and scr-silenced HE6F cells (Fig. 5c). The percentages of apoptotic measured as the Annexin/PI double positive population were 21% for Axl-silenced HE6F compared with 10% for scrambled siRNA (Scr si)-silenced HE6F cells after sFASL stimulation. Consistently, proapoptotic genes, such as Fas, TRADD, and FADD, were markedly decreased in Axl-silenced HE6F compared with the levels in the mock control (Fig. 5d). In CaSki cells, the proliferation and invasion of tumour cells were increased by treatment with an Axl antibody as an agonist (Supplementary Fig. S6). These data suggested that Axl expression induced by E6 plays a role in the malignant tumorigenicity of cervical cancer.

### The expression of Axl and MZF1 is correlated with clinical stage of cervical cancer and HPV16/18 infection

**Figure 6:**
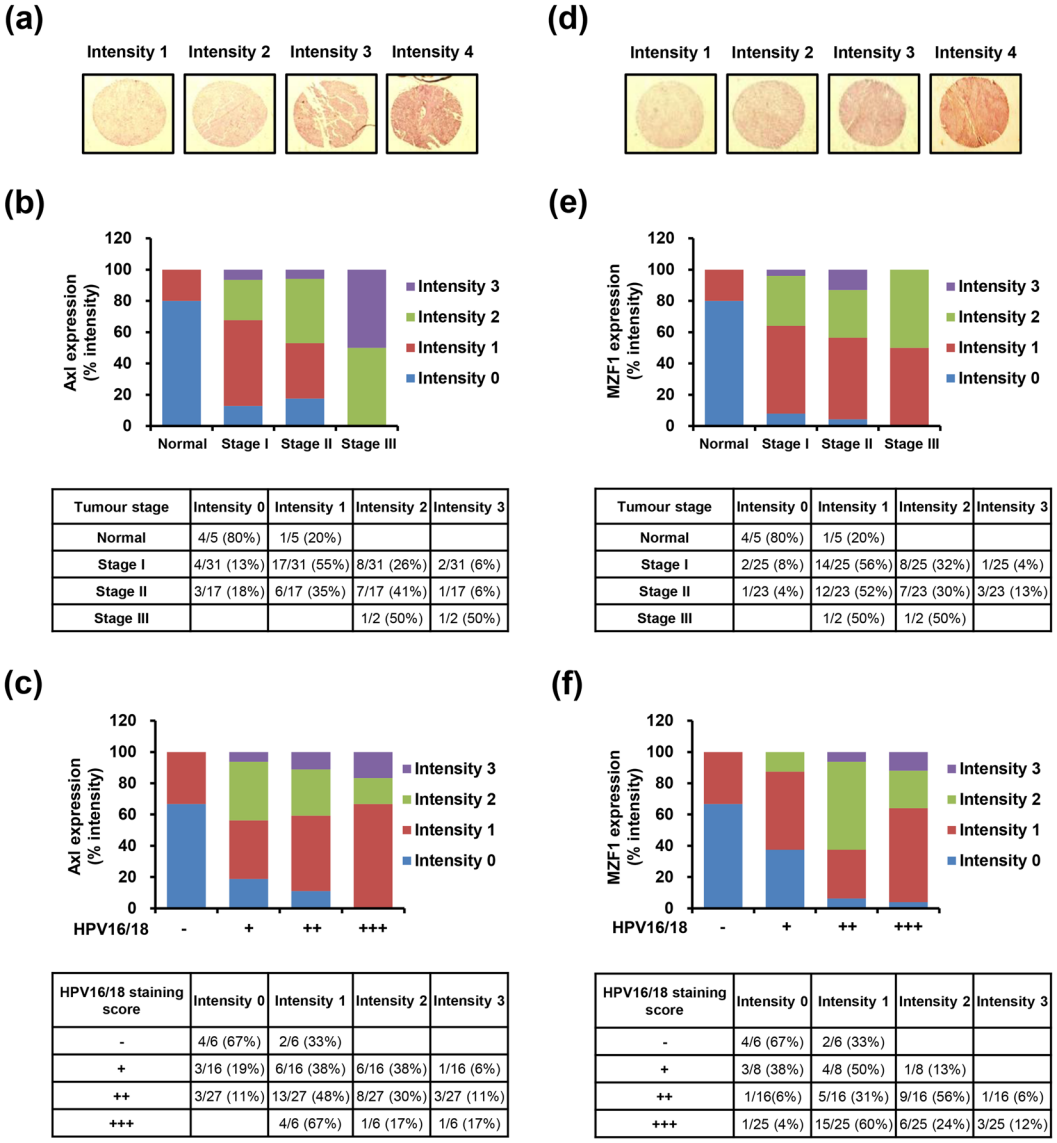
Correlation of the expression of Axl and MZF1 with clinical stage of cervical cancer and degree of HPV16/18 infection. (**a**) Representative images of normal and cervical cancer tissues with different IHC intensities positive for Axl. (**b**) The relative percentage of IHC intensity in normal and tumour stage-specific tissues was calculated from data of table, and Axl expression was represented as a bar graph. (**c**) Axl expression dependent on degree of HPV16/18 infection. (**d**) Representative images of IHC intensities positive for MZF1. (**e**) The intensities of MZF1 expression. (**f**) MZF1 expression dependent on degree of HPV16/18 infection. Normal: No evidence of primary tumor, Stage I: Tumor invades submucosa, Stage II: Tumor invades muscularis propria, Stage III: Tumor invades through muscularis propria into subserosa or into non-peritonealized pericolic or perirectal tissues. Staining scores for HPV16/18: “−” no staining; “+” weak staining; “++” moderate staining; “+++” strong staining.

To elucidate the association of Axl and MZF1 overexpression with the progression of cervical cancer and degree of HPV16/18 infection, we assessed the expression of Axl and MZF1 in both normal human cervix tissues from 10 individuals and cervical cancer tissues from 100 different patients by immunohistochemistry using tissue microarray. The immunohistochemical expression of Axl and MZF1 in the tissues was represented as intensity scores and representative images of intensity 0 (weak), 1 (moderate), 2 (strong) and 3 (very strong) were shown in Fig. 6a and d. The expression of Axl was gradually increased in cervical cancer tissues in a tumour stage-dependent manner compared with normal tissues (Fig. 6b). Moreover, the expression pattern of Axl was also dependent on degree of HPV infection (Fig. 6c). Similarly to the expression of Axl, the expression levels of MZF1 were also much higher in cervical cancer than in normal tissues in tumour stage- and degree of HPV16/18 infection-dependent manners (Fig. 6e and f). These data suggest that the expression of Axl and MZF1 was correlated with clinical stage of cervical cancer and HPV 16/18 infection.

## Discussion

Although the Axl receptor tyrosine kinase is known to be involved in the malignancy of various cancers^28^, and high-risk HPV-positive cervical cells express Axl^19^, the molecular mechanisms that upregulate Axl expression in response to the high-risk HPV16E6 oncogene are unclear. In this study, we found that the E6 oncoprotein of HPV16 upregulated *Axl* gene expression, resulting in an increase in Axl protein levels (Fig. 1).

A vast number of cellular targets for the E6 and E7 oncogenes have been identified^29–31^. In HPV-induced malignancy, both of these oncogenes are potential targets for a therapeutic HPV vaccine. The exact mechanisms by which E6 exerts its transforming activity remain to be investigated; however, it seems that the ability of E6 to target cell polarity regulators may be key. A large number of PDZ domain-containing proteins in regions of cell-cell contact or tight junctions are targets of the E6 oncogene. However, to improve cervical cancer control, studies of the downstream events regulated by the HPV E6 oncogene that target cell polarity regulators are required. Our results showed that ectopic expression of HPV16E6 in HeLa cells enhanced Axl expression and that suppression of HPV16E6 by antisense or siRNA inhibited the expression of Axl, which indicated that HPV16E6 enhances *Axl* gene expression. Furthermore, silencing of HPV16E6 by antisense RNA suppressed the AKT phosphorylation in HE6F, CaSki, and Sihs cells (Fig. 3b and c). Interestingly, MAGI-2 expression and PTEN phosphorylation (S380) induced by HPV16E6 knockdown were increased in those cervical cancer cells (Fig. 3). Here, we clarified a new pathway, showing that E6-mediated Axl expression is triggered by the MAGI-2/PTEN/AKT signalling pathway.

The previous study has reported that Axl promoter activity is reduced by mutation in the most important motif (−479/−473) for Axl promoter activation^19^. However, the motif is overlapped with secondary major transcription start site (SMTSS). It was therefore speculated that the motif might not be the most relevant site in transcriptional activity of Axl. Moreover, they has used translation start site (TSS, +1) of Axl promoter region, but we used transcription start site (+1) according to the predicted site as described in Methods. Thus, SMTSS in Reference #19 corresponds with TSS in the present study. In addition, they has examined MZF1 binding sites such as −563/−557, −479/−473 and −80/−72 regions, but not −514/−505 region. In the present study, deletion of −47/−33 region (−522/−508 in Reference #19) resulted in reduced Axl promoter activity (Fig. 2a), and mutation of −37/−32 region (−512/−507 in Reference #19) also led to no binding activity of MZF1 in CaSki cells (Fig. 2c). We also found the binding activity of MZF1 to Axl promoter as determined by ChIP assay (Fig. 2d). Thus, we showed, for the first time, that motif at −39/−30 (−514/−505 in Reference #19) on Axl promoter was the most functionally relevant site as a binding motif for MZF1.

Axl is overexpressed in several human cancer cell lines and tumour samples, and Axl expression has been correlated with advanced tumour stage. Gas6/Axl signalling promotes the growth, survival, and proliferation of numerous cell types through activation of the RAS/RAF/MAPK/ERK1/2 and PI3K signalling pathways. Recent study have demonstrated that the MZF1 transcription factor induced Axl expression in solid cancers^19^; however, the precise mechanisms involved are unclear. In this study, we revealed that HPV16E6-mediated Axl expression was MZF1 dependent. Our present observation provides the evidence that the HPV16E6 oncoprotein upregulates Axl expression through the MAGI-2/PTEN/AKT/MZF1 signalling pathway. In addition, HPV16E6-mediated Axl increases tumour invasiveness and immune evasion by decreasing susceptibility to NK-mediated lysis, demonstrating that upregulation of Axl expression plays an important role in cervical cancer tumorigenicity. The expression of Axl and MZF1 was highly associated with clinical stage and degree of HPV16/18 infection in human cervical cancer tissues. Our data collectively suggest that HPV16E6 may act as a key positive regulator of Axl expression in cervical carcinomas, and its regulation in malignant cervix squamous lesions may be an effective way to achieve stable suppression of HPV16E6 tumorigenesis.

## Methods

### Cell lines, antibodies, and reagents

HeLa (HPV18-positive), CaSki (HPV16-positive), Siha (HPV16-positive) human cervical cancer cells and 293 T human embryonic kidney cells were obtained from the American Type Culture Collection (ATCC; Manassas, VA, USA). HeLa, CaSki, and Siha cells were cultured in α-MEM supplemented with 10% foetal bovine serum (FBS) and antibiotics, at 37 °C in a humidified 5% CO_2_ incubator, and 293 T cells were maintained in DMEM culture medium. Western blotting and immunocytofluorescence analysis were conducted using the following antibodies: anti-Axl, anti-MZF1, anti-Sp1, and anti-LaminlB1 (Santa Cruz Biotechnology, Santa Cruz, CA, USA); anti-HPV16E6 (Full Moon Biosystems, Sunnyvale, CA, USA); anti-AKT and anti-p-AKT (Cell Signaling Technology, Beverly, MA, USA); anti-PTEN and anti-p-PTEN (R&D Systems, Abingdon, UK); anti-MAGI-2 (Novus Biologicals, Littleton, CO, USA); and α-tubulin (Sigma, St. Louis, MO, USA). Horse radish peroxidase (HRP)-conjugated anti-goat IgG and anti-rabbit IgG (Santa Cruz Biotechnology) were used as secondary antibodies. Soluble Fas ligand (sofas L) was purchased from Enzo life science (CA, USA).

### DNA constructs

DNA constructs were generated as described previously^21^. The pcDNA3.1 mammalian expression vector was used to express HPV16E6 in HeLa and CaSki cells. HPV16E6 DNA was obtained from the genomic DNA of CaSki cells. The cDNA for HPV16E6 was cloned into a pcDNA3.1 expression vector containing the neomycin resistance gene to construct two plasmids expressing HPV16E6 sense RNA (sense HPV16E6 plasmid) or HPV16E6 antisense RNA (antisense HPV16E6 plasmid), depending on the orientation of the insert. The sequences of all constructs were confirmed by DNA sequencing.

### Cell transfection

HeLa cells (5–10 × 10^6^ cells) that did not express HPV16E6 were transfected with either the plasmid vector or sense HPV16E6 plasmid (5 μg), and CaSki cells that overexpressed HPV16E6 were transfected with either the plasmid vector or antisense HPV16E6 plasmid using Lipofectamine 2000 according to the manufacturer’s instructions (Invitrogen, Carlsbad, CA, USA). To obtain stable transfected clones, after 48 h of incubation, the cells were cultured in individual medium containing 1.0 mg/mL of G418 (Sigma) for 2 weeks. Then, G418-resistant cells were selected by limiting dilution for single cells. The surviving clones were harvested, transferred to 35-mm^2^ culture dishes, and grown in medium without G418. Stable HeLa transfectants expressing HPV16E6 were named HE6F, and stable CaSki transfectants suppressing HPV16E6 were named CE6R.

### Small interfering RNA

Small interfering RNA (siRNA) experiments were performed by transfecting cells with siRNA oligomers encoding HPV16E6, Axl, MZF1, or MAGI-2. The HE6F and Axl siRNA oligomers were obtained from Samchully Pharmaceuticals (Seoul, Korea). Scrambled, MZF1, and MAGI-2 siRNA oligomers were purchased from Santa Cruz Biotechnology. Briefly, 200 pmol of siRNA was mixed with Lipofectamine 2000 (Invitrogen) and transfected them into HeLa and CaSki cells. At 4 h post-transfection, the siRNA-containing medium was replaced with normal complete culture medium, and cells were incubated at 37 °C in 5% CO_2_. After 48 h of incubation, cells were harvested for RT-PCR and western blotting. The siRNA sequences were as follows: HPV16E6, 5′-CCAUAUGCUGUAUGUGAUATT-3′ and 5′-UAUCACAUA CAGCAUAUGGTT-3′; Axl #1, 5′-GAG UAC CAA GAG AUU CAU ATT-3′ and 5′-UAU GAA UCU CUU GGU ACU CTT-3′; and Axl #2, 5′-ACA GCG AGA UUU AUG ACU ATT-3′ and 5′-UAG UCA UAA AUC UCG CUG UTT-3′.

### Semiquantitative RT-PCR and quantitative real-time PCR

Semiquantitative RT-PCR and quantitative real-time PCR were carried out as described previously^32^. The primers used for PCR are listed in Supplementary Table 1.

### Matrigel invasion assay

A Matrigel invasion assay was performed with BioCoat Matrigel invasion chambers (Becton Dickinson Labware, Bedford, MA, USA) according to the manufacturer’s protocol. Briefly, 3 × 10^5^ cells were resuspended in 100 μL of serum-free medium and placed in the upper compartment of the transwell invasion chamber (8 μm pores). To the lower compartment of the chamber, 800 μL of α-MEM containing 10% FBS was added as a chemoattractant. After incubation for 36 h at 37 °C in 5% CO_2_, cells that did not invade the filter were wiped away with a cotton swab, while the cells that had invaded the lower surface of the filter were fixed with 100% methanol. The cells were then stained with hematoxylin and eosin (Sigma), and counted in five randomly selected microscopic fields (100×) per filter.

### Western blot analysis

Cells were washed twice with cold PBS and lysed in protein lysis buffer (10 mM Tris-HCl, pH 7.4, 150 mM NaCl, 2 mM EDTA, 1% Nonidet P-40, 1 mM PMSF, 10 μg/mL aprotinin, and 10 μg/mL leupeptin) for 30 min on ice. After centrifugation of the lysates, the protein concentration was determined by Bradford assay according to manufacturer’s instructions (Bio-Rad). Cell lysates containing equal amounts of protein (100 μg) were separated by 10–15% SDS-PAGE and transferred to a polyvinylidene fluoride membrane (Millipore, Marlborough, MA, USA). The blot was incubated with blocking buffer (1% BSA and 5% skim milk in PBS) overnight at 4 °C, and then washed three times with TBST (50 mM Tris. pH 7.4, 150 mM NaCl, 0.05% Tween 20). The blot was incubated with the indicated primary antibodies for 2 h at room temperature, and then incubated with HRP-conjugated secondary antibodies for 1 h at room temperature. After three washings in TBS, the signal was detected with the ECL system (GE Healthcare, Marlborough, MA, USA). To re-probe the blot with other antibodies, the membranes were incubated in stripping buffer (62.5 mM Tris-HCl buffer, pH 6.7, 100 mM mercaptoethanol, and 2% SDS) with shaking for 30 min at 55 °C. After three washings with TBST, western blotting was performed as described above.

### Cytotoxicity assay of NK cells

The cytotoxic activity of NK cells was measured by a lactate dehydrogenase (LDH)-release assay according to the manufacturer’s instructions (Promega, Madison, WI, USA). For the NK cytotoxicity assay, NK92mi cells were utilized as effector cells, and cervical cancer cells were used as target cells for measuring cytolytic activity. The effector cells were mixed with target cells (1 × 10^4^) at different effector-to-target (E:T) ratios (100:1–1:1) in a 96-well U-bottom plate, and incubated for 4 h at 37 °C. Fifty microliters of culture supernatants were then collected from each well and tested for LDH activity using a chromogenic substrate. Absorbance was measured at 495 nm using an ELISA reader (Bio-Rad), and the percent specific lysis was determined by the following formula: (experimental effector spontaneous release − experimental target spontaneous release)/(target maximum release − target spontaneous release) × 100.

### Deletion mutants in the human Axl promoter and luciferase reporter assay

Deletion constructs of the Axl promoter were generated, and a luciferase assay was performed as described previously^33^. Promoter prediction was performed using promoter database online program (https://cb.utdallas.edu/cgi-bin/TRED/tred.cgi?process=home). Analysis of potential transcriptional factor binding sites was carried out using TFSEARCH online programs (http://diyhpl.us/~bryan/irc/protocol-online/protocol-cache/TFSEARCH.Html). The primers used for the Axl promoter deletion constructs are listed in Supplementary Table 1. For the luciferase assay, 293 T cells were cotransfected with a pGL3-luciferase vector containing various *Axl* promoters and a pcDNA3.1 plasmid encoding HPV16E6. Cells were lysed in luciferase assay lysis buffer (BD Biosciences/PharMingen, San Jose, CA, USA), and luciferase activity was measured with enhanced chemiluminescence reagents (BD Biosciences/PharMingen) in a Monolight 2010 luminometer (Analytical Luminescence, San Diego, CA, USA). Luciferase activity was measured, and the transfection efficiency was normalized to beta-galactosidase activity.

### Electrophoretic mobility shift assay (EMSA) and ChIP assay

Nuclear extracts were prepared as described previously^33^. Briefly, 10 μg of nuclear extracts was incubated with a [γ-^32^P]dATP-labelled double-stranded oligonucleotide containing MZF1 or OCT-1 binding motifs for 30 min at room temperature. Then, the mixtures were electrophoresed on a 6% polyacrylamide gel and analysed by autoradiography. The sequences of the oligonucleotides are as follows: MZF1 consensus oligos, 5′-AGTGGGGACGGGGAGGGGGAA-3′; OCT-1, 5′-TGTCGAATGCAAATCACTAGAA-3′; MZF1 WT oligo (−37/−3 bp), 5′-GGCGGGGAGAGGGGCGTCACGGCCTGAGCTGA GAG-3′; MZF1 Mutant oligo, 5′-GAATTTGAGAGGGGCGTCACGGCCTGAGCTGA GAG-3′. To perform ChIP assay, cells were cross-linked by formaldehyde and resuspended in SDS lysis buffer followed by sonication to shear genomic DNA. Immunoprecipitation was performed by adding anti-MZF1 or normal IgG control. After addition of protein G agarose beads, co-precipitated DNA fragments were eluted. Recovered DNA was purified by QIAquick PCR purification kit (Qiagen) and the purified DNA was used as PCR template. PCR primers for ChIP assays were as follows: Forward: 5′-TGG GGG TGG AGG CGG GGA-3′; Reverse: 5′-CAG TGA GGC CGT GTC TCT CT-3′. Quantification of MZF1-binding (% input) was performed by determining the amount of specific band compared with input DNA from three independent experiments.

### Annexin V/PI assays for apoptosis

For the annexin V/PI assays, cells were stained with annexin V-FITC and PI, and evaluated for apoptosis by flow cytometry according to the manufacturer’s protocol (BD PharMingen). Briefly, 1 × 10^6^ cells were washed twice with phosphate-buffered saline (PBS) and stained with 5 µL of annexin V-FITC and 10 µL of PI (5 µg/mL) in 1× binding buffer (10 mM HEPES, pH 7.4, 140 mM NaOH, and 2.5 mM CaCl_2_) for 15 min at room temperature in the dark. Apoptotic cells were determined using a Becton-Dickinson FACScan cytoflurometer (Mansfield, MA, USA). Both early (annexin V-positive, PI-negative) and late (annexin V-positive and PI-positive) apoptotic cells were included in the cell death determinations.

### Immunofluorescence analysis

CE6R cells were fixed in 4% paraformaldehyde in PBS for 15 min. After three washes with PBS, the cells were permeabilized for 10 min with 0.1% Triton X-100 in PBS and then blocked with 5% BSA for 1 h. Cells were incubated overnight at 4 °C with PTEN antibody (1: 1000 diluted) and MAGI-2 antibody (1: 500 diluted). Finally, the primary antibodies were revealed by means of appropriate AlexaFluor 555 conjugates for PTEN or AlexaFluor 488 conjugates for MAGI-2. After counterstaining with DAPI (4, 6-diamidino-2-phenylindole), immunofluorescense images were captured with Olympus IX71 fluorescence microscope (Olympus, Tokyo, Japan).

### Immunohistochemical staining and tissue microarray

Tissue microarrays (CR1101) (US Biomax, Rockville, MD) were baked for 1 h at 60 °C before deparaffinization and rehydration, and sections were boiled in antigen retrieval citrate buffer (pH 6.0) for 30 min. Primary antibodies were incubated for overnight and the sections were further incubated for 1 h with HRP-conjugated anti-mouse or goat secondary antibody followed by a diaminobenzidine (DAB) kit as substrate for HRP. After counterstaining of the sections with hematoxylin and staining for Axl and MZF1 immunostaining, individual intensities of immunoreactivity were classified as a 0–3 rating scale by Image J computer program; intensity 0 (weak), 1 (moderate), 2 (strong) and 3 (very strong).

### Statistical analysis

Significance was calculated using Student’s *t*-test. *P* values less than 0.05 were considered statistically significant. All experiments were performed at least five times independently.

## Electronic supplementary material

Supplementary Information
