# Supplementary material for: Blockade of Axl signaling ameliorates HPV16E6-mediated tumorigenecity of cervical cancer
Source: Sci Rep. 2017 Jul 18;7:5759. doi: 10.1038/s41598-017-05977-8 (PMC5516033; doi:10.1038/s41598-017-05977-8)
Supplement: Supplementary file 1 — Supplementary Information [file 41598_2017_5977_MOESM1_ESM.pdf]

Supplementary Figures

Blockade of Axl signaling ameliorates HPV16E6-mediated tumorigenecity of cervical cancer

Eun-Hee Lee, Kon-Young Ji, Eun-Mi Kim, Su-Man Kim, Hyeong-Woo Song, Ha-Rim Choi, Byung Yeoup Chung, Hyo Jin Choi, Hyoung-Woo Bai, and Hyung-Sik Kang

Contents:

Figure S1
Figure S2
Figure S3
Figure S4
Figure S5
Figure S6
Table 1

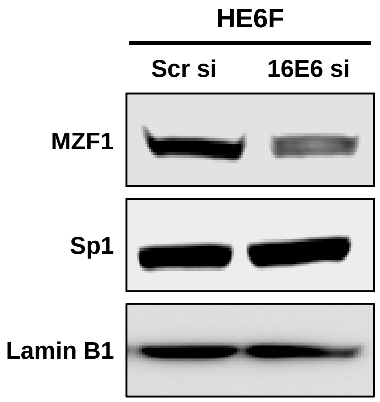

Figure S1. HPV16E6 suppressed the nuclear localization of MZF1 transcription factor. The expression of MZF1 was decreased in the nuclear fraction of HE6F cells by transfection of E6 siRNA. The expression of Sp-1 was not altered.

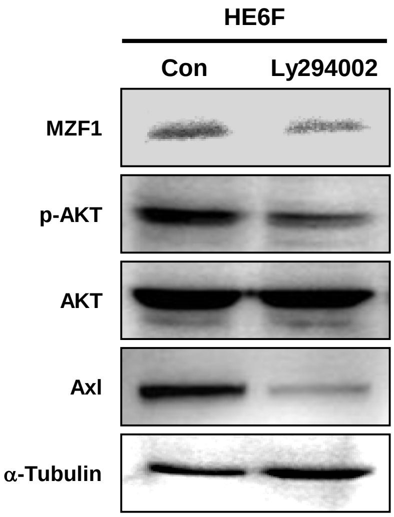

Figure S2. AKT signalling is involved in HPV16E6-induced Axl expression. Protein expression of Axl was analysed by western blotting in HE6F cells treated with10μM Ly294002 for 24 h. Western blots showing that the levels of phosphorylated Akt and MZF1 were decreased.

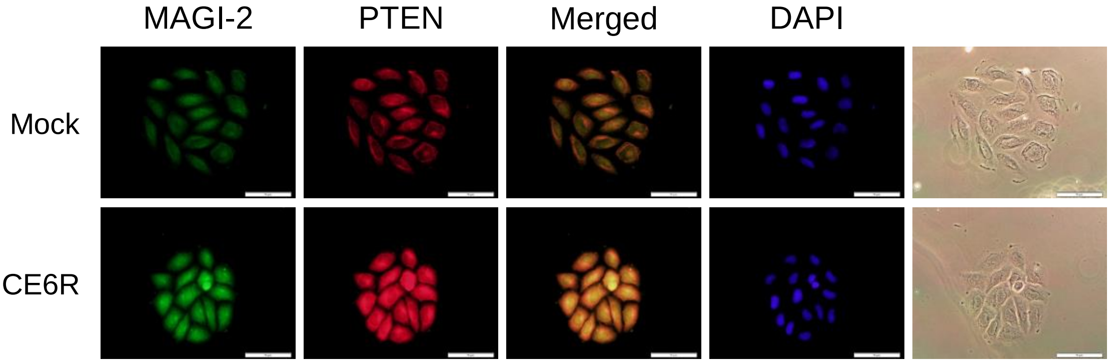

Figure S3. Co-localization of MAGI-2 and PTEN in mock control and CE6R cells. Subcellular localization of endogenous MAGI-2 (green) and PTEN (red) was analyzed by fluorescence microscopy in mock control and CE6R cells. The yellow signal in the merged images represented an overlapping spatial relationship between green and red fluorescence. Scale bar = 50 μm. Data are representative of three independent experiments.

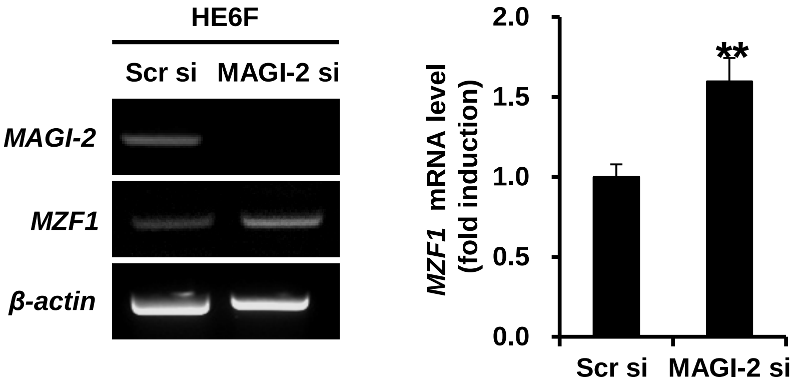

Figure S4. Upregulation of MZF1 by silencing of MAGI-2 in HE6F cells. MZF1 mRNA expression levels were determined by quantitative RT-PCR in HE6F cells transfected with MAGI-2 siRNA (left panel). After quantitation of the individual bands from qRT-PCR using densitometry, mRNA expression levels of MZF1 were calculated as a fold change compared with scramble siRNA (Scr si), and represented as bar graph (right). Data represent the mean ± standard deviation of triplicate determinations (**, $P < 0.01$).

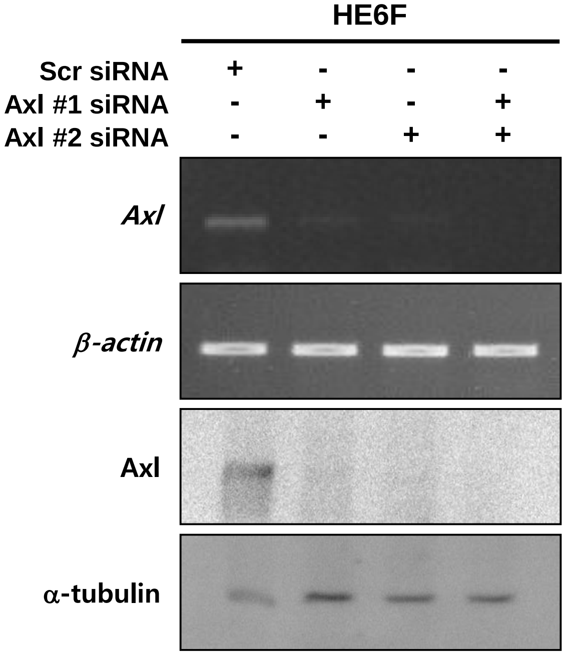

Figure S5. Effect of siRNA on silencing Axl gene transcription and protein expression. Representative pictures of RT-PCR and western blot experiments showing expression of mRNA and protein for Axl. HE6F cells were transfected with 50 nM of two siRNA(Axl#1 and Axl#2) for 24 h. The mock group was transfected in the scrambled RNA.

(a)

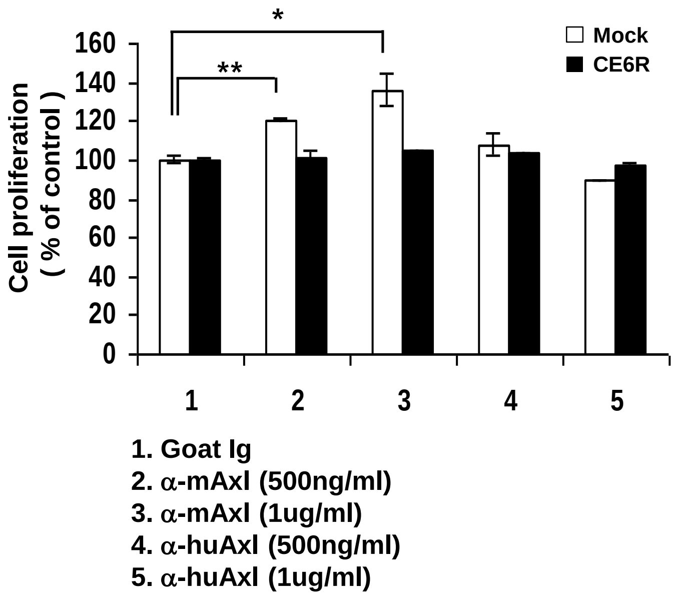

(b)

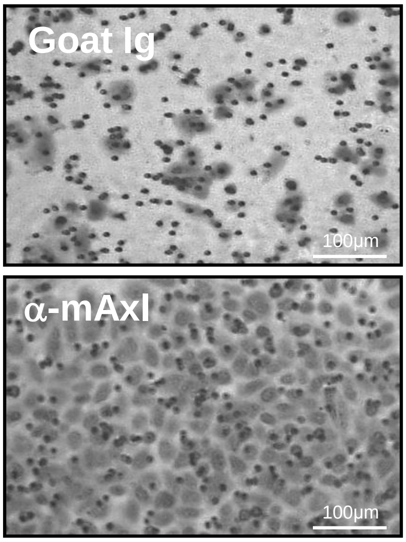

Figure S6. Effect of siRNA on silencing Axl gene transcription and protein expression. Tumorigenicity of CaSki cells was increased by stimulation of Axl signalling. (**a**) Proliferation of CE6R cells after treatment with an anti-mAxl as an agonistic effectors or anti-hAxl as an antagonistic effectors, was determined by MTS assay. The levels were calculated as a percentage change relative to the mock and CE6R group treated with Goat Ig and the data are presented as the mean ± standard deviation of three independent experiments. *P<0.05 (**b**) CaSki cells stimulated with anti-Axl (1 mg/mL) showed increased invasiveness.

Supplementary Table 1. Primers used in RT-PCR, real-time PCR and cloning

**Primers for RT-PCR**		
**Gene**	**Sense (5'-3')**	**Antisense (5'-3')**
HPV 16 E6	GGG GAA TTC TAT AGT GTG TAT GGA ACA	GGG GAA TTC TTA CAG CTG GGT TTC TCT
Axl	CCC AGA ACC TGT GGT CAT CT	ACC GAG ACA TCA GGG CAT AC
MAGI-2	GGC AGT ACC CTC TGT CGG AC	CCC CTG GCC GCC GGG CGC CT
Fas	ACT TGG GGT GGC TTT GTC TT	GGA TGA TAG TCT GAA TTT TCT CTG
TRADD	CAG CAG AAG GTG GCA GTG TA	GCT TCA CCT CCG ACA GAG AG
FADD	CTG CCT TGG CAA TTC TGT TAT CAG	TGG CTG GGG TGG GGG TGG GGA GAC
MZF1	CGG GCG AAC AGC CTT TCC GT	GGT GAG CCG GAC CTG CG
β-actin	GTG GGG CGC CCC AGG CAC CA	CTC CTT AAT GTC ACG CAC GAT TTC
**Primers for Real-time PCR**		
**Gene**	**Sense (5'-3')**	**Antisense (5'-3')**
Axl	CGT CCG TGT GCT GGA TGA	TCC CAT CGT CTG ACA GCA
**Primers for cloning of deletion constructs of LIGHT promoter**		
**Gene**	**Sense (5'-3')**	**Antisense (5'-3')**
Axl (401)-luc	TCC CAT TTA GGC GTC CAT G	CGG ATA GAG AGA CAC GGC CT
Axl (351)-luc	CCCCAAGTAAGTGTCCCCCATGG	CGG ATA GAG AGA CAC GGC CT
Axl (201)-luc	CTG CTT GTC CTA GCC TGT G	CGG ATA GAG AGA CAC GGC CT
Axl (81)-luc	TGA AGG GCC AAG GAG GCC TG	CGG ATA GAG AGA CAC GGC CT
Axl (47)-luc	TGG GGG TGG AGG CGG GGA GA G	CGG ATA GAG AGA CAC GGC CT
Axl (33)-luc	GGG AGA GGG GCG TCA CGG CC	CGG ATA GAG AGA CAC GGC CT
